# Supplementary material for: Development of a Potential Yeast-Based Vaccine Platform for Theileria parva Infection in Cattle
Source: Front Immunol. 2021 Jul 8;12:674484. doi: 10.3389/fimmu.2021.674484 (PMC8297500; doi:10.3389/fimmu.2021.674484)
Supplement: Supplementary file 1 [file Image_1.pdf]

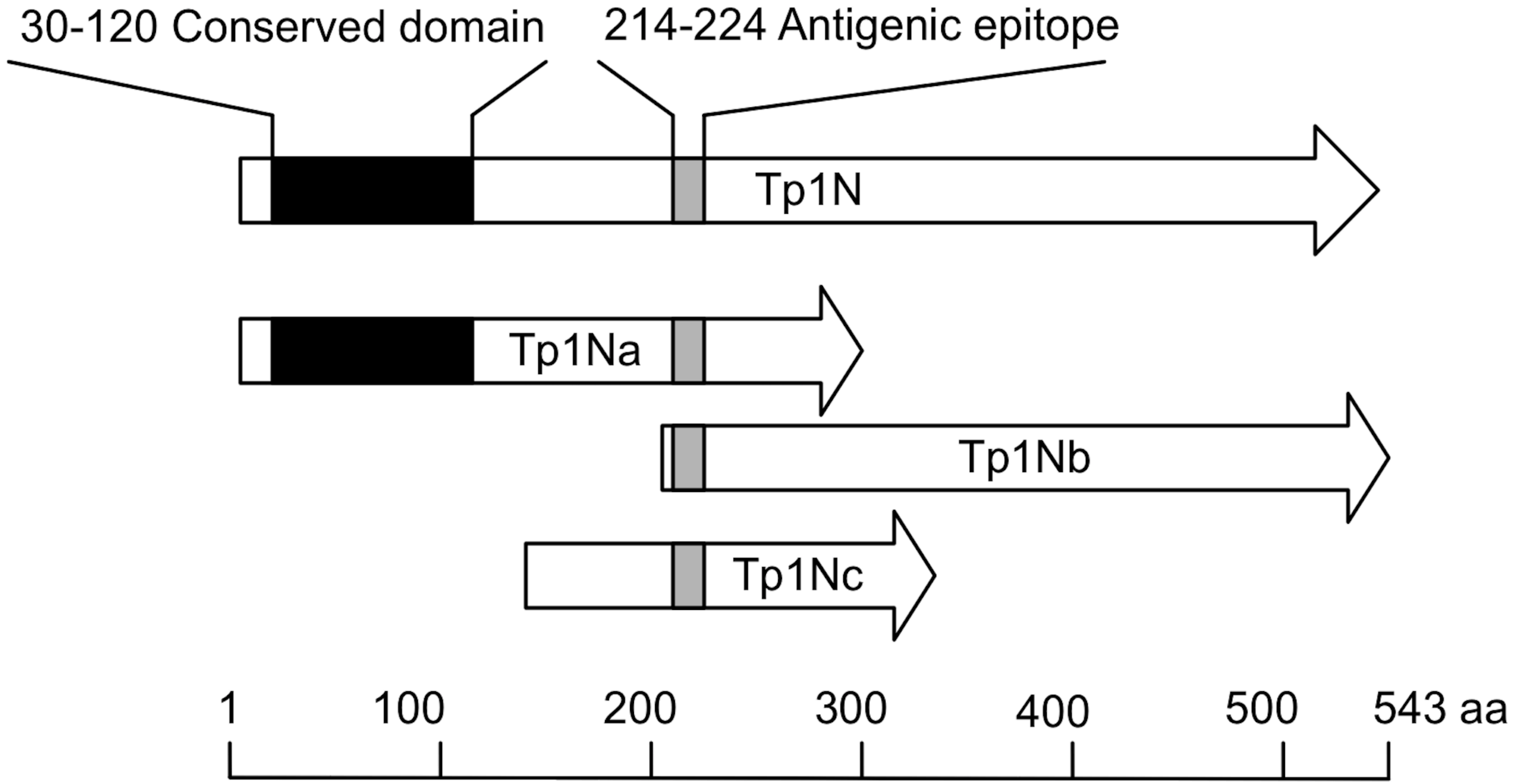

Supplementary Figure 1 : Genetic subunits of native Tp1 (Tp1N) expressed in pYD1. Known features of native Tp1 are indicated and the antigenic CD8^{+} T cell epitope is present in all subunits.
