# Supplementary material for: Development of a Potential Yeast-Based Vaccine Platform for Theileria parva Infection in Cattle
Source: Front Immunol. 2021 Jul 8;12:674484. doi: 10.3389/fimmu.2021.674484 (PMC8297500; doi:10.3389/fimmu.2021.674484)
Supplement: Supplementary file 4 [file Image_4.pdf]

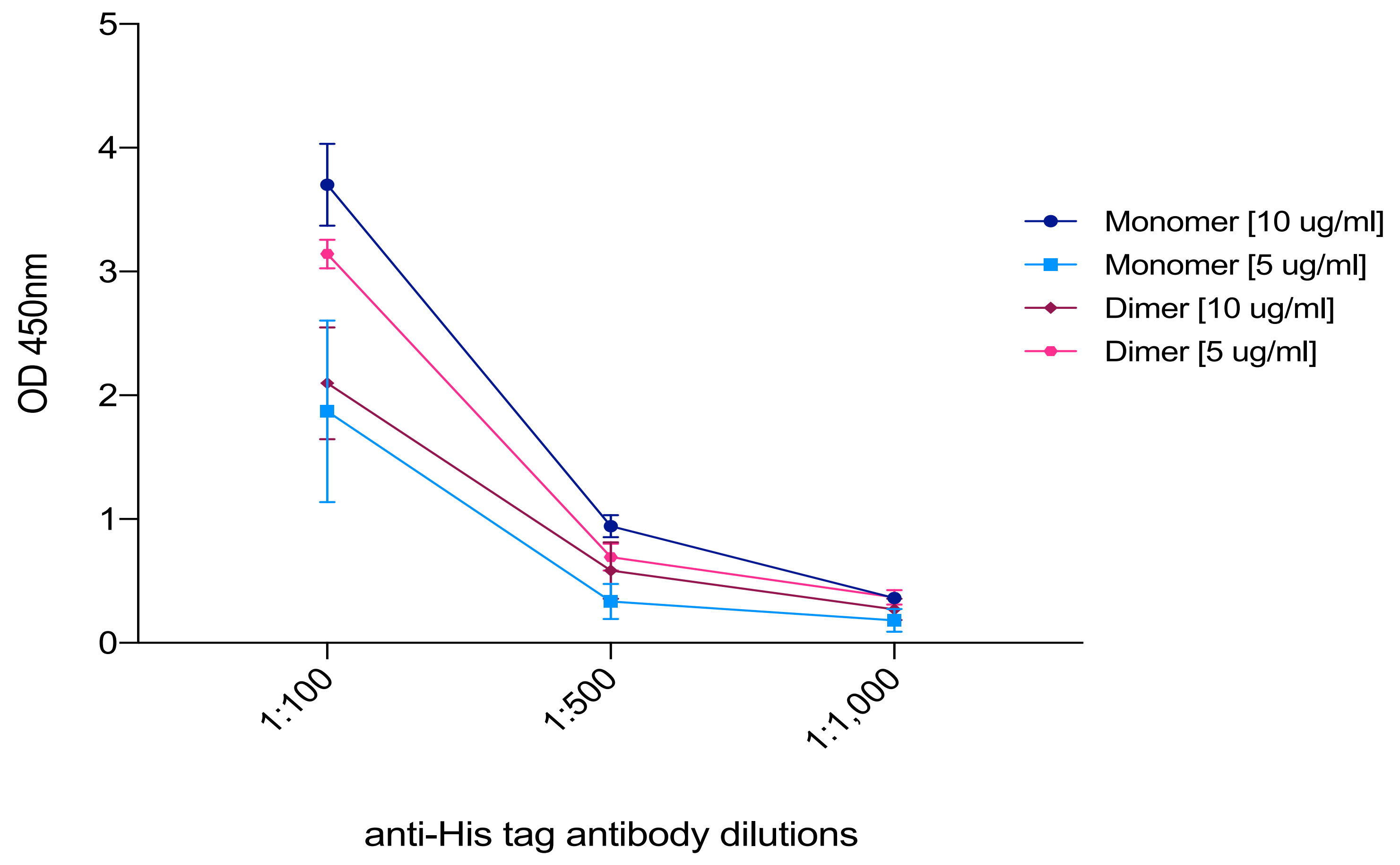

Supplementary Figure 4: Detection of Tp2 expressed by *E. coli* BL21 Star on Nunc MaxiSorp™ Flat bottom 96-well ELISA plates after overnight incubation at 4°C. Binding of both monomer and dimer fractions (10 µg ml^{-1} and 5 µg ml^{-1}) was evaluated with three dilutions of an anti-Histidine tag monoclonal antibody. Each fraction was detected by the antibody in a dose-dependent manner confirming proper binding of the protein to the plate. However, lower concentrations of dimer resulted in a stronger signal compared to higher concentrations. Consequently, the monomer was selected for further experiments. The results displayed represent one of two independent experiments.
