# Supplementary material for: Development of a Potential Yeast-Based Vaccine Platform for Theileria parva Infection in Cattle
Source: Front Immunol. 2021 Jul 8;12:674484. doi: 10.3389/fimmu.2021.674484 (PMC8297500; doi:10.3389/fimmu.2021.674484)
Supplement: Supplementary file 5 [file Table_1.docx]

Supplementary Table 1: Comparison of pYD1 and pYES2/NTC expression vectors

| Features | pYD1 plasmid | pYES2/NTC plasmid |
| --- | --- | --- |
| Empty plasmid length (bp) | 5,009 | 6,037 |
| Origin of replication | CEN6/ARS4 | 2μ |
| Largest expressed protein (published) | 53 kD (BVDV E2) | 130 kD (CEA) |
| Plasmid localisation | Episomal | Episomal |
| Plasmid copy number | 1-2 | 10-40 |
| Recombinant protein localisation | Surface | Intracellular |
| Expression control | Inducible | Inducible |
| Expression host | EBY100 | INVSc1 |
| Auxotrophic selection of mutants | Tryptophan | Uracil |
